# Prediction of global omicron pandemic using ARIMA, MLR, and Prophet models

**DOI:** 10.1038/s41598-022-23154-4

**Published:** 2022-10-28

**Authors:** Daren Zhao, Ruihua Zhang, Huiwu Zhang, Sizhang He

**Affiliations:** 1 Department of Medical Administration, Sichuan Provincial Orthopedics Hospital, Chengdu, 610041 Sichuan China; 2 School of Management, Chengdu University of Traditional Chinese Medicine, Chengdu, 611130 Sichuan China; 3 Department of Information and Statistics, The Affiliated Hospital of Southwest Medical University, Luzhou, 64600 Sichuan China

**Keywords:** Epidemiology, Preventive medicine

## Abstract

Globally, since the outbreak of the Omicron variant in November 2021, the number of confirmed cases of COVID-19 has continued to increase, posing a tremendous challenge to the prevention and control of this infectious disease in many countries. The global daily confirmed cases of COVID-19 between November 1, 2021, and February 17, 2022, were used as a database for modeling, and the ARIMA, MLR, and Prophet models were developed and compared. The prediction performance was evaluated using mean absolute error (MAE), mean absolute percentage error (MAPE), and root mean square error (RMSE). The study showed that ARIMA (7, 1, 0) was the optimum model, and the MAE, MAPE, and RMSE values were lower than those of the MLR and Prophet models in terms of fitting performance and forecasting performance. The ARIMA model had superior prediction performance compared to the MLR and Prophet models. In real-world research, an appropriate prediction model should be selected based on the characteristics of the data and the sample size, which is essential for obtaining more accurate predictions of infectious disease incidence.

## Introduction

Since November 2021, the Omicron variant has rapidly spread worldwide. The B.1.1.529 variant was first reported by WHO in South Africa on November 24, 2021^1^. The World Health Organization (WHO) announced the SARS-CoV-2 variant Omicron (B.1.1.529) on November 26, 2021^2,3^. Consequently, many countries have enacted various restrictions to prevent the spread of Omicron variants.

Globally, as of February 14, 2022, a total of 416,614,051 confirmed cases of COVID-19 comprising 5,844,097 deaths were reported by the WHO^4^. It was estimated that the *R*_*0*_ of the Omicron variants may be as high as 10^5^. Therefore, it is crucial that prediction models are used to forecast the COVID-19 epidemic trend, which can help the government and relevant authorities take effective measures to respond in advance^6^. Time series forecasting models play an important role in disease surveillance^7^. Accurate prediction results are required for the prevention and control of COVID-19 to provide early warning information to government officials.

Numerous mathematical models, including traditional time series and machine learning models, have been applied to predict the incidence of COVID-19. In particular, in the traditional time series model, the ARIMA time series model is the most widely used for COVID-19 incidence prediction. Ceylan et al.^8^ used the ARIMA model to estimate the overall prevalence of COVID-19 in three European countries, and the results can help politics and health authorities allocate medical resources reasonably. Sun et al.^9^ used a modified ARIMA model to forecast the COVID-19 pandemic in Alberta, Canada. Roy et al.^10^ analyzed the effectiveness of COVID-19 epidemiological surveillance using ARIMA models. Malki et al.^11^ applied the ARIMA model to predict the spread of COVID-19 worldwide. James et al.^12^ adopted the ARIMA model to forecast the short-term trajectory of the acceleration of fatalities caused by COVID-19. Dawoud et al.^13^ utilized the ARIMA model to estimate COVID-19 cumulative confirmed cases. Alzahrani et al.^14^ used the autoregressive model (AR), moving average (MA), a combination of both (ARMA), and integrated ARMA (ARIMA) to forecast the COVID-19 pandemic and found that the performance of the ARIMA model outperformed the other models.

In addition, the ARIMA model is used not only in the estimation of the number of COVID-19 pandemics, but also in the estimation of the number of fully vaccinated people or in the estimation of electricity consumption and natural gas amounts. Cihan et al.^15^ developed the ARIMA model to predict electricity and natural gas consumption in an industrial zone in Turkey. Cihan et al.^16^ used the ARIMA model to determine the number of people fully vaccinated against COVID-19.

However, some of the research has focused on the use of machine learning models to predict COVID-19 incidence, such as LSTM, GRU, SVR, XGBoost, RNNs, etc. Shahid et al.^17^ constructed the ARIMA, SVR, LSTM, and Bi-LSTM models to forecast COVID-19 confirmed cases, deaths, and recoveries in ten major countries, and stated that Bi-LSTM achieved much better prediction results than other models. Luo et al.^18^ established and compared the prediction performance of the LSTM and XGBoost algorithms. ArunKumar et al.^19^ developed GRU, LSTM, and RNN models to forecast future trends of the cumulative COVID-19 confirmed cases for the top-10 countries.

However, to date, no studies have compared global COVID-19 incidence predictions using ARIMA, MLR, and Prophet models since the outbreak of Omicron variants. In this study, the global daily confirmed cases of COVID-19 between November 1, 2021, and February 17, 2022, were obtained from the WHO website. Based on the sample size and data characteristics, ARIMA, MLR, and Prophet models were constructed and compared, and the optimum model was selected to predict the global daily confirmed cases of COVID-19 from February 18 to March 18, 2022. To the best of our knowledge, this is the first study to explore in detail the construction and comparison of the ARIMA, MLR, and Prophet models for predicting daily confirmed cases of COVID-19 worldwide. We hope that the prediction results of this study will serve as a reference for COVID-19 prevention and control worldwide.

## Materials and methods

### Materials

#### Data source

We collected daily confirmed cases of COVID-19 globally between November 1, 2021, and February 17, 2022, from the website of the World Health Organization (https://covid19.who.int/). Microsoft Excel was used to create the time series database. All data were updated daily. In this study, 109 observations were divided into training and validation sets, 80% of which was the training set, and the rest (20%) was the test set. The datasets for November 1, 2021, and January 27, 2022, were considered as the training set, and data from January 28, 2022, to February 17, 2022, were considered as the validation set.

### Methods

#### ARIMA model

The autoregressive integrated moving average (ARIMA) model, a classic time series prediction technique, was proposed by Box and Jenkins in the early 1970s, and has been extensively applied to the prediction of infectious diseases^20^. ARIMA is a mathematical model that uses historical values to forecast future values of a variable^21^. The basic equation for ARIMA is as follows^22^:

1 $$ \Theta_{P} (B^{s} )\theta_{p} (B)(1 - B^{s} )^{D} (1 - B)^{d} y_{t} = \Phi_{Q} (B^{s} )\varphi_{q} (B)\varepsilon_{t} $$

In this equation, *y*_t_ is the predictive value, *B* is the backward shift operator, *ε*_t_ is the residuals from time series^23^,$\Theta_{P}$ and $\theta_{p}$, $\Phi_{Q}$, and $\varphi_{q}$ represent the four parameters in the ARIMA model *p*, *q*, *P*, and *Q*, respectively. Here, *d* and *D* represent the degrees of the seasonal and trend differences, respectively. The ARIMA model parameters *p*, *P*, *q*, *Q*, and *s* represent the order of auto-regression, seasonal auto-regression lag, order of moving average, seasonal moving average, and seasonal periodicity, respectively^24^.

In general, the ARIMA model is defined as ARIMA(*p*, *d*, *q*) (*P*, *D*, *Q*)s. In this study, however, the ARIMA model was expressed as ARIMA(*p*, *d*, *q*) because the daily confirmed COVID-19 cases in the time series were non-seasonal data, and its equation can be written as follows^23^:

2 $$ \theta_{p} (B)(1 - B)^{d} y_{t} = \varphi_{q} (B)\varepsilon_{t} $$

The construction process of the ARIMA model includes several steps^25–28^. First, the daily confirmed COVID-19 case sequence was plotted to determine whether the time series was stationary. Sequences with non-stationary time series were transformed into stationary sequences using difference and log transformations. Second, the parameters of the ARIMA model were estimated by analyzing auto-correlation and partial auto-correlation function graphs. The parameters *p*, *P*, *q*, and *Q* were determined using auto-correlation function (ACF) and partial auto-correlation function (PACF) graphs after difference and log transformations. The candidate ARIMA model was determined initially. Third, the ARIMA model diagnosis and evaluation were determined using the Ljung-Box (Q) test and the t-test, respectively. The Ljung-Box (Q) test required that residuals of the daily COVID-19 case time series were white noise (significant level, *p* > 0.05). A t-test was used to determine whether the parameters of each candidate ARIMA model were significant. The optimum model depends on the maximum R-square value, minimum normalized BIC, and RMSE values, and the residuals are white noise sequences. Bayesian information criterion (BIC) is commonly used for model selection in time series forecasting^29^. It was developed by Schwarz and is defined as^29,30^:

3 $$ {\text{BIC}} = { - }2\ln (L) + \ln (n)*k $$

where *L* is the maximized value of the likelihood function of the model, *n* is the sample size, and *k* is the number of parameters estimated by the model. The normalized Bayesian information criterion (BIC) was used to confirm the adequacy of the model^30^. The smaller the value of the normalized BIC, the more adequate the model fits^30^.

#### MLR model

Multiple linear regression model(MLR), an extension of simple linear regression, is used to describe the a linear relationship between multiple independent variables and a single dependent variable^31^. The formula for the MLR model is as given below^32^.

4 $$ {\text{Y}} = \beta + \beta_{0} {\text{X}}_{1} + \beta_{1} {\text{X}}_{2} + ... + \beta_{k} {\text{X}}_{k} + \varepsilon $$

where *Y* is the dependent variable; *X*_1_, *X*_2_, … are the independent variables; *β* is the *Y*-intercept; *β*_0_, *β*_1_,… *β*_k_ are the regression coefficients; and ε is the random error term.

#### Prophet model

The Prophet model, an open-source time-series forecasting algorithm, was created by Facebook in 2017, and can be run using R or Python^33^. The basic formula for the Prophet model is as follows^34,35^:

5 $$ y(t) = g(t) + s(t) + h(t) + \varepsilon_{t} $$

Here, *y*_*(t)*_ is the predictive value, *g(t)* is the trend function that models non-periodic changes in the time series of daily confirmed COVID-19 cases, *s (t)* signifies periodic changes(weekly characteristics of confirmed COVID-19 cases time series), and *h (t)* signifies the effects of holidays on potentially irregular schedules. For example, Christmas Day. $\varepsilon_{t}$ signifies idiosyncratic changes that are not accommodated by the model^36^.

In trend model *g (t)*, there are two types of models: a saturating growth model and a one-piece linear model that covers numerous Facebook applications. The formula for the nonlinear saturation growth model is as follows^37^:

6 $$ g(t) = \frac{{\text{C}}}{1 + \exp ( - k(t - m))} $$

where *C* is the carrying capacity, *k* is the growth rate, and *m* is the offset parameter.

The formula for the piecewise logistic growth model is as follows^36^:

7 $$ g(t) = \frac{{\text{C(t)}}}{{1 + \exp ( - (k + \alpha (t)^{T} \delta )(t - (m + \alpha (t)^{T} \gamma )))}} $$

where $\delta$ is a vector of rate adjustments and $\gamma$ is the correct adjustment at the change point.

The seasonality *s(t)* depends on the Fourier series to provide a viable model for periodic effects. This formula is expressed as follows^34^.

8 $$ s(t) = \sum\limits_{n = 1}^{N} {\left( {a_{n} \cos \left( {\frac{2\pi nt}{P}} \right) + b_{n} \sin \left( {\frac{2\pi nt}{P}} \right)} \right)} $$

where *a* is standard Fourier series, *P* is the periodic changes.

Holidays and events *h(t)* have a greater influence on predicting time-series performance because they do not follow a periodic pattern^37^.

9 $$ {\text{Z}}(t) = \left[ {1(t \in D_{1} ,...1(t \in D_{L} )} \right] $$

10 $$ {\text{h}}(t) = Z(t)k $$

where *t* is during holiday *i* and *ki* is the holiday parameter and a prior *k* ~ normal (0, ν^2^).

#### Evaluation of the prediction performance

 In this study, the mean absolute error (MAE), mean absolute percentage error (MAPE), and root mean square error (RMSE) were used to evaluate the prediction performances of the ARIMA, MLR, and Prophet models. The smaller the values of MAE, MAPE, and RMSE, the better is the prediction performance of the model. These evaluation indices are expressed as^38^:

11 $$ {\text{MAE}} = \frac{{\sum\limits_{t = 1}^{n} {\left| {X_{t} - {\hat{\text{X}}}_{t} } \right|} }}{n} $$

12 $$ {\text{MAPE}} = \frac{{\sum\limits_{t = 1}^{n} {\left| {\frac{{X_{t} - {\hat{\text{X}}}_{t} }}{{X_{t} }}} \right| \times 100{\text{\% }}} }}{n} $$

13 $$ {\text{RMSE}} = \sqrt {\frac{{\sum\limits_{t = 1}^{n} {(X_{t} - {\hat{\text{X}}}_{t} )^{2} } }}{n}} $$

where ${\hat{\text{X}}}_{t}$ is the predicted value, $X_{t}$ is the observed value, and n is the sequence sample size.

#### Statistical software

 SPSS (version 24.0; IBM Corp., Armonk, NY, USA, URL: https://www.ibm.com/support/pages/node/724325?mhsrc=ibmsearch_a&mhq=statistics%2024) and EView (version10.0; IHS Global Inc., Irvine, CA, USA, URL:https://eviews.com/download/ev10download.shtml) were used to create the ARIMA model. SPSS version 24.0 (version 24.0; IBM Corp., Armonk, NY, USA, URL: https://www.ibm.com/support/pages/node/724325?mhsrc=ibmsearch_a&mhq=statistics%2024) was used to create the MLR model. R software (version 4.1.1, URL:https://stat.ethz.ch/pipermail/r-announce/2021/000672.html) was used to construct the Prophet model. Among which, “Prophet” package of R software was used in construction of the Prophet model. The level of significance was set at *p* < 0.05.

### Ethical approval

Data were obtained from publicly accessible sources. Formal ethical approval was not required for this study.

## Results

### General analysis

**Figure.1:**
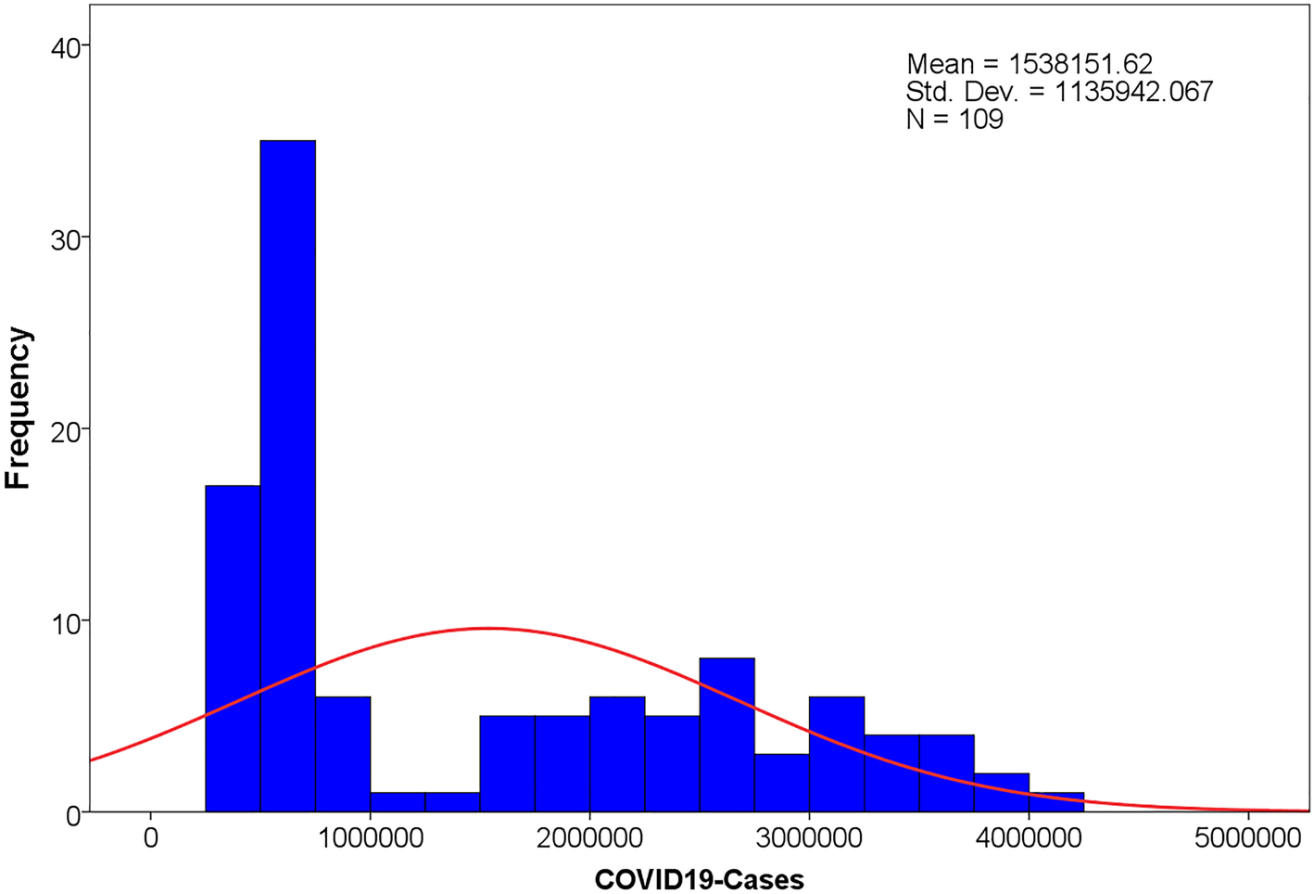
The histogram chart of the daily confirmed cases of COVID-19.

**Figure.2:**
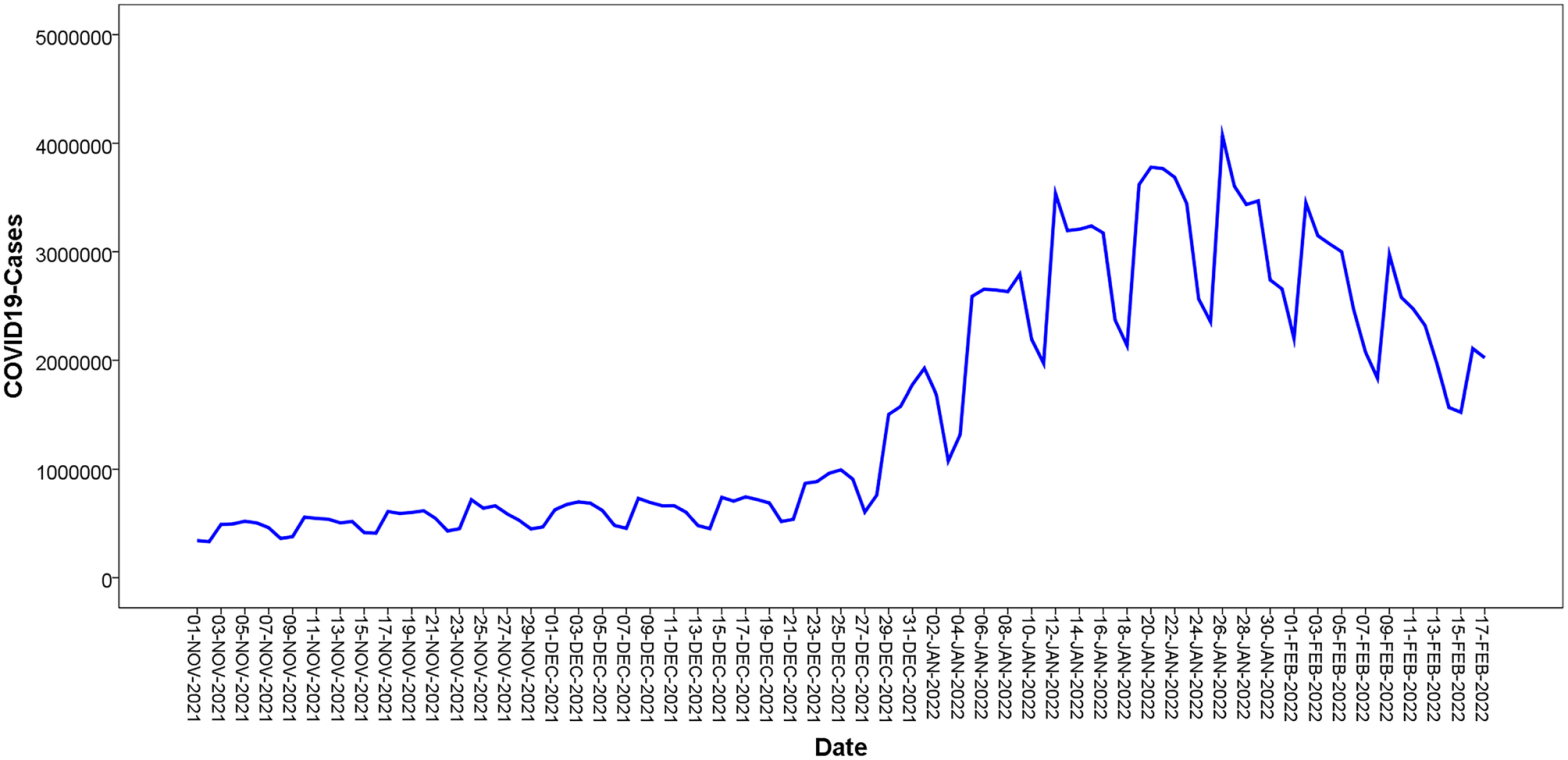
The original sequence chart of the daily confirmed cases of COVID-19 time series.

A total of 167,658,527 confirmed cases of COVID-19 were reported worldwide between November 1, 2021, and February 27, 2022. Descriptive Statistics of the daily confirmed cases of COVID-19 are shown in Table 1. The histogram chart of the daily confirmed cases of COVID-19 is shown in Fig. 1. As shown in Fig. 2, there was a rising periodicity trend characteristic of the daily confirmed cases of the COVID-19 time series. The growth rate of new confirmed coronary cases was 1.92% per day during this period. In addition, the confirmed cases occurred at a minimum peak on the first day and then reached a high peak two days later every other week with a cycle of 7 days (Fig. 2).

**Table 1:** Descriptive Statistics of the daily confirmed cases of COVID-19.

Indicators	Mean	Median	Std. dev	Minimum	Maximum	Skewness	Kurtosis
Statistics	1,538,151.62	886,342	1,135,942.07	332,100	4,068,855	0.64	− 1.08

### ARIMA model

**Figure.3:**
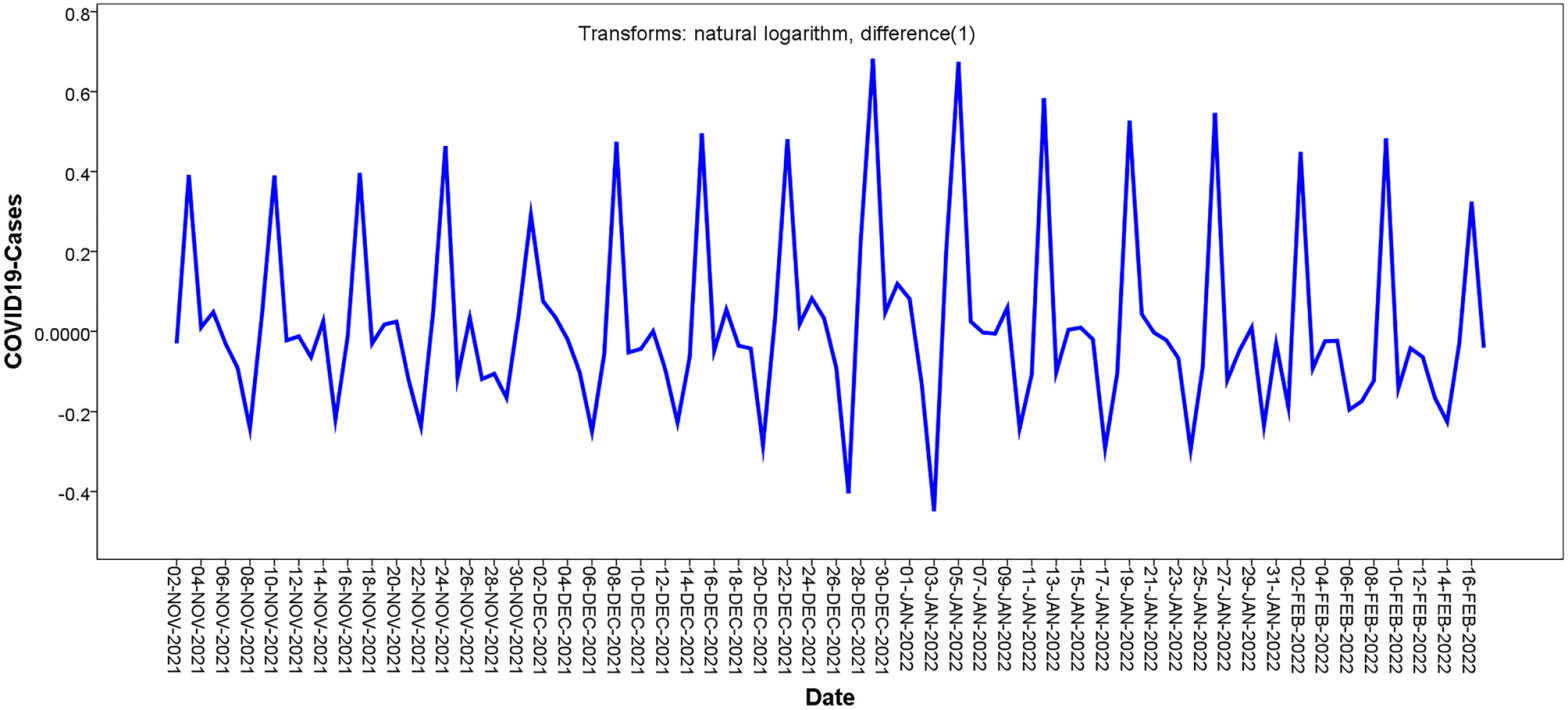
Time series chart of after the first-order difference and natural logarithm transformation.

The original sequence of the daily confirmed cases of the COVID-19 time series fluctuated greatly and presented an upward and periodic trend, indicating that this was a non-stationary time series (Fig. 2). Therefore, we used the first-order difference and natural logarithm transformation to convert the original sequence into a stationary time series; thus, parameter d was 1. The transformed time series presented random and stable characteristics (Fig. 3) and was a stationary time series. The ADF test also showed that the transformed time series was stationary ( *t* = − 9.247, *p* < 0.001).

**Figure.4:**
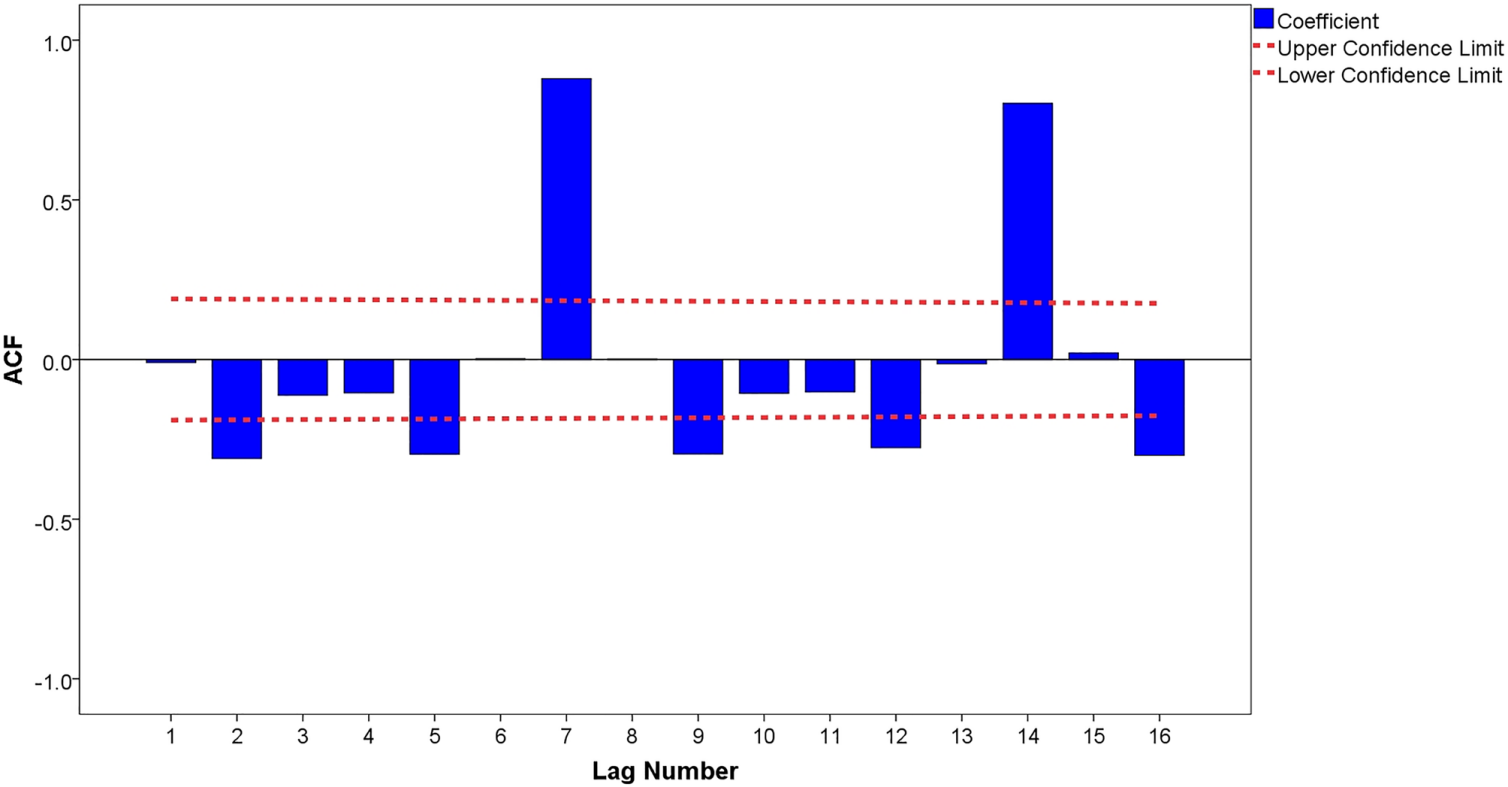
ACF chart of after the first-order difference and natural logarithm transformation.

**Figure.5:**
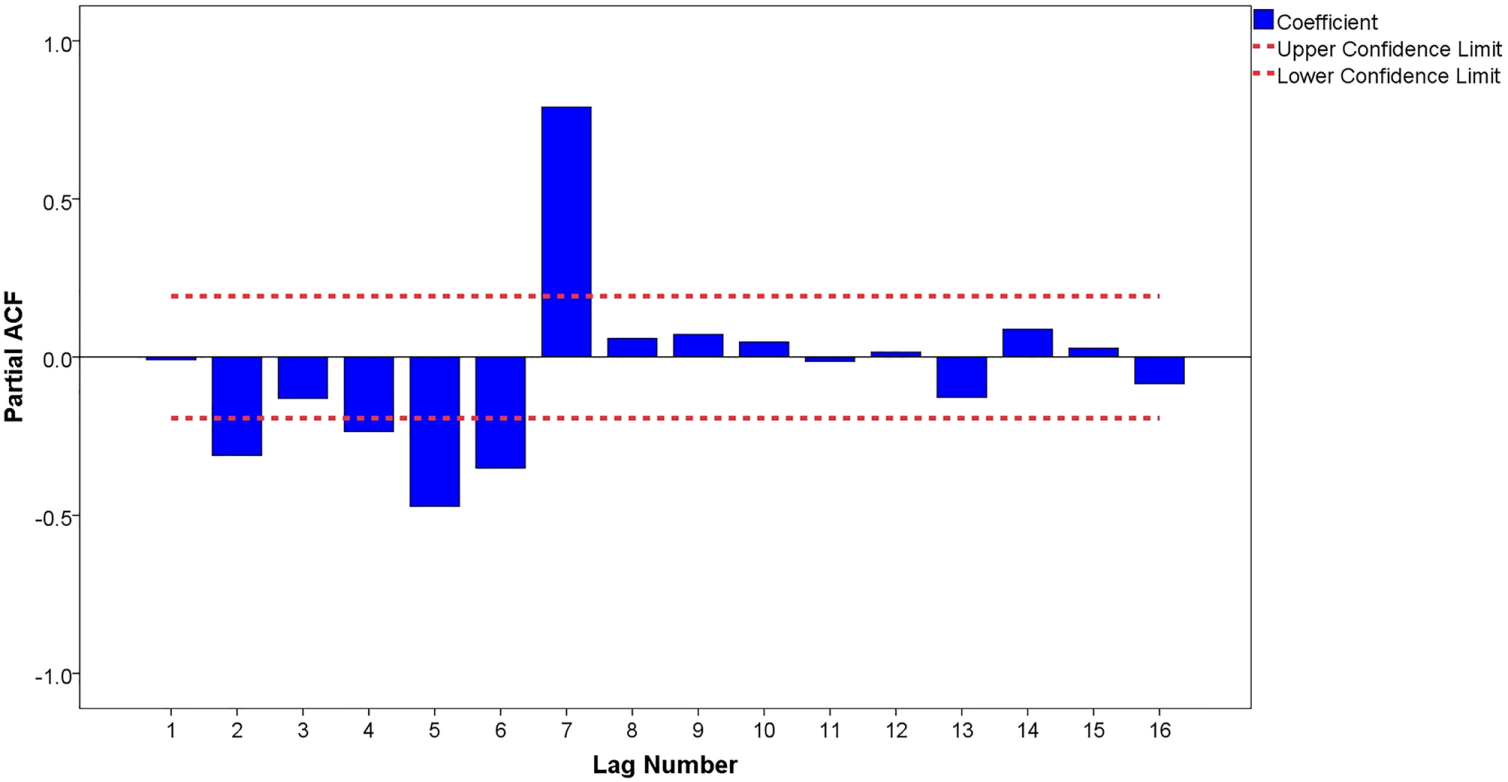
PACF chart of after the first-order difference and natural logarithm transformation.

The autocorrelation function (ACF) and partial autocorrelation function (PACF) graphs can help identify the *p*, *q*, *P*, and *Q* parameters of the ARIMA model. The candidate ARIMA models were constructed by combining the parameters *p*, *q*, *P*, and *Q*. From the analysis in Figs. 4, 5, we found that after a first-order difference and natural logarithm transformed time series displayed trailing and slower decaying convergence, the maximum was on the order of 7, which was significantly higher than orders 1 to 6; therefore, the parameter of *p* was 7, and *q* was in the range of 0 to 7. Therefore, the candidate ARIMA models are as follows: ARIMA (7,1,0), ARIMA (7,1,1), ARIMA (7,1,2), ARIMA (7,1,3), ARIMA (7,1,4), ARIMA (7,1,5), ARIMA (7,1,6), and ARIMA (7,1,7).

**Figure.6:**
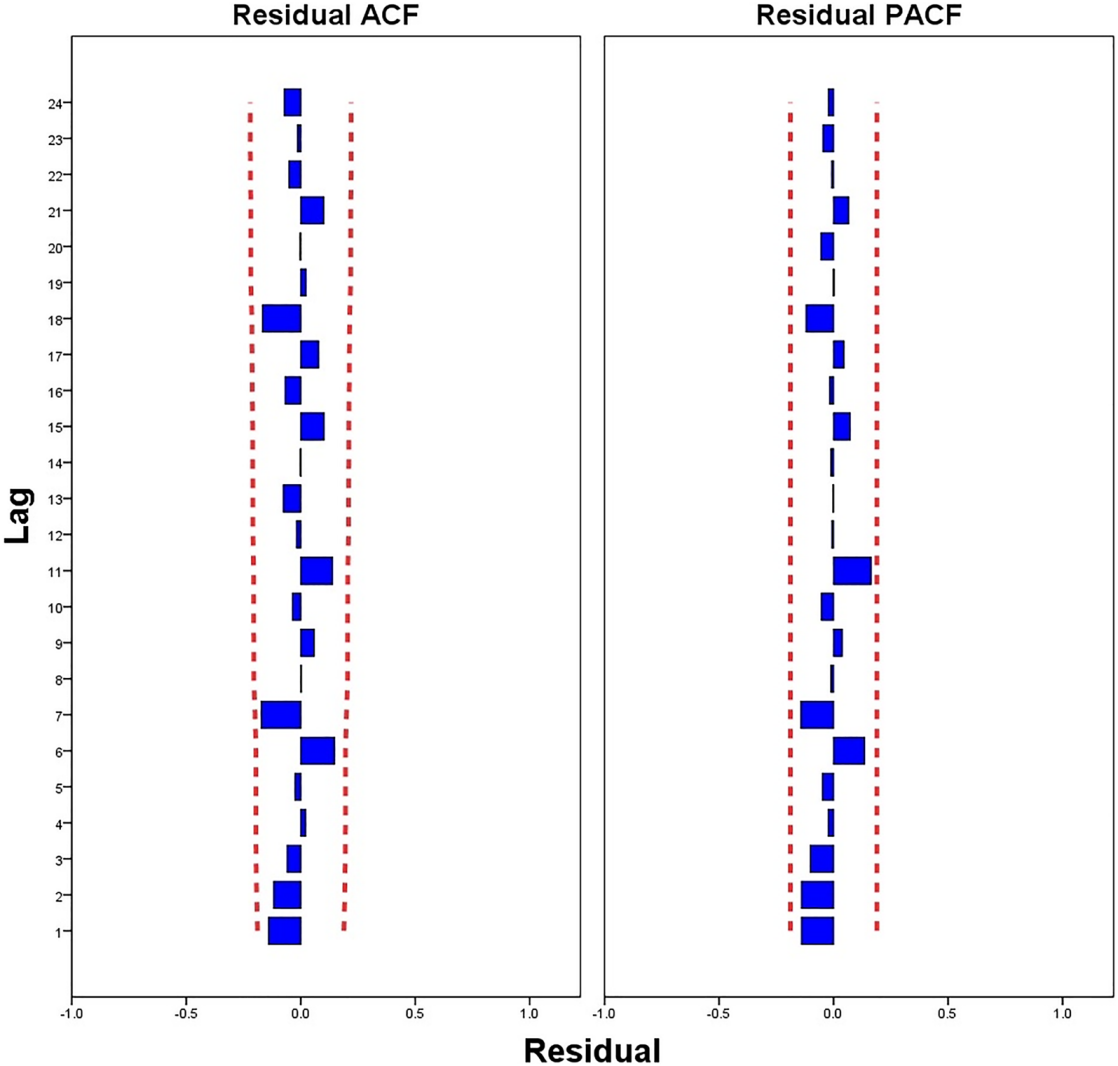
The residual ACF and PACF chart of the ARIMA(7,1,0) model.

In addition, all candidate ARIMA models were tested using Ljung-Box Q for white noise. The results show that only three models passed the Ljung-Box Q test(*p* > 0.05): ARIMA(7,1,0), ARIMA(7,1,1), and ARIMA(7,1,2) (Table 2). The larger the R-squared value, the better is the fit of the ARIMA model. As shown in Table 2, the difference between the R squared values of the three models was not significant, indicating that the degree of the fitting effect was not different. Furthermore, we found that ARIMA(7,1,0) had the lowest RMSE and normalized BIC values and passed the t-test(*p* < 0.001), indicating that it was the optimum model (Table 3). Figure 6 shows that the residual ACF and PACF charts of ARIMA(7,1,0) are stationary time series, which also demonstrates that ARIMA(7,1,0) is the optimum model.

**Table 2:** Parameter estimation of the candidate ARIMA models.

Candidate models	R-squared	RMSE	Normalized BIC	Ljung-box Q(18)		
				Statistics	DF	*p* value
ARIMA (7,1,0)	0.975	178,064.179	24.223	20.223	17	0.256
ARIMA (7,1,1)	0.977	179,911.555	24.591	16.132	10	0.096
ARIMA (7,1,2)	0.977	179,852.547	24.633	15.817	9	0.071

**Table 3:** Estimates and standard error of three candidate ARIMA models.

Candidate model			Estimate	SE	*t*	*p* value
ARIMA(7,1,0)	AR	Lag 7	0.900	0.037	24.515	0.000
	Difference		1			
ARIMA(7,1,1)	AR	Lag 7	0.866	0.048	17.956	0.000
	Difference		1			
	MA	Lag 1	0.298	0.117	2.552	0.012
ARIMA(7,1,2)	AR	Lag 7	0.876	0.048	18.173	0.000
	Difference		1			
	MA	Lag 1	0.251	0.122	2.059	0.042
	MA	Lag 2	0.098	0.122	0.800	0.426

### MLR model

The confirmed cases occurred at a minimum peak on the first day and then reached a high peak two days later every other week with a cycle of 7 days. Therefore, the every6th moment(day) might have affected the values at the latter moment(day). We used the data sliding method to set the input variables (*X*_1_-*X*_6_) and the independent variable(*Y*) and then constructed a multiple linear regression model. The R value of the MLR model was 0.949, indicating that the model fit well. The results of the F-test showed that the linear regression equation was significant (*F* = 144.08, *p* < 0.05). The MLR model equation that we fitted was ${\text{Y}} = 90416.43 + 0.4{\text{X}}_{1} - 0.1{\text{X}}_{2} + 0.02{\text{X}}_{3} + 0.01{\text{X}}_{4} - 0.16{\text{X}}_{5} + 0.71{\text{X}}_{6}$. The results are shown in Table 4.

**Table 4:** The parameters of MLR model.

Model	Unstandardized coefficients		Standardized coefficients	*t*	*p* value
	B	Std. error	Beta		
Constant	90,416.43	63,838.996		1.416	0.16
*X*_*1*_	0.40	0.10	0.41	4.19	0.00
*X*_*2*_	− 0.10	0.12	− 0.10	− 0.81	0.42
*X*_*3*_	0.02	0.12	0.02	0.14	0.89
*X*_*4*_	0.10	0.12	0.10	0.83	0.41
*X*_*5*_	− 0.16	0.12	− 0.16	− 1.30	0.20
*X*_*6*_	0.71	0.10	0.71	7.45	0.00

### Prophet model

**Figure.7:**
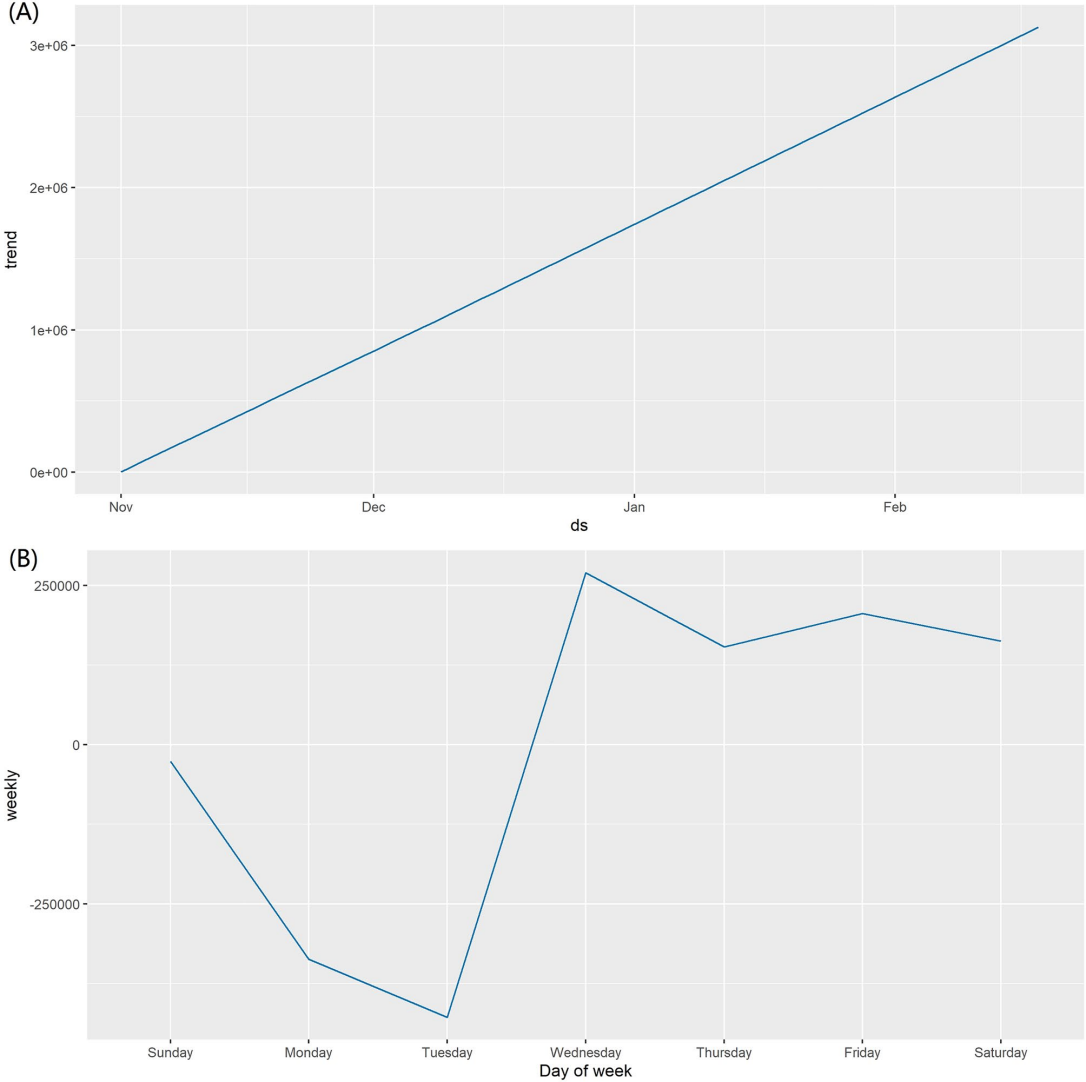
The decomposed components of the daily confirmed cases of COVID-19 time series. (**A**) the trend, (**B**) the weekly.

A total of 109 observations are included in this section. In this study, the Prophet model that we constructed excluded holidays because of the differences in holidays between countries and the rapid spread of Omicron variants worldwide. As shown in Fig. 7, the global daily confirmed cases of the COVID-19 time series showed a fast-growing upward trend between November 2021 and February 2022. The day of the week curve shows that the global daily confirmed cases of the COVID-19 time series dropped to their lowest point on Tuesdays, quickly reached their highest point on Wednesdays, and then gradually increased.

**Figure.8:**
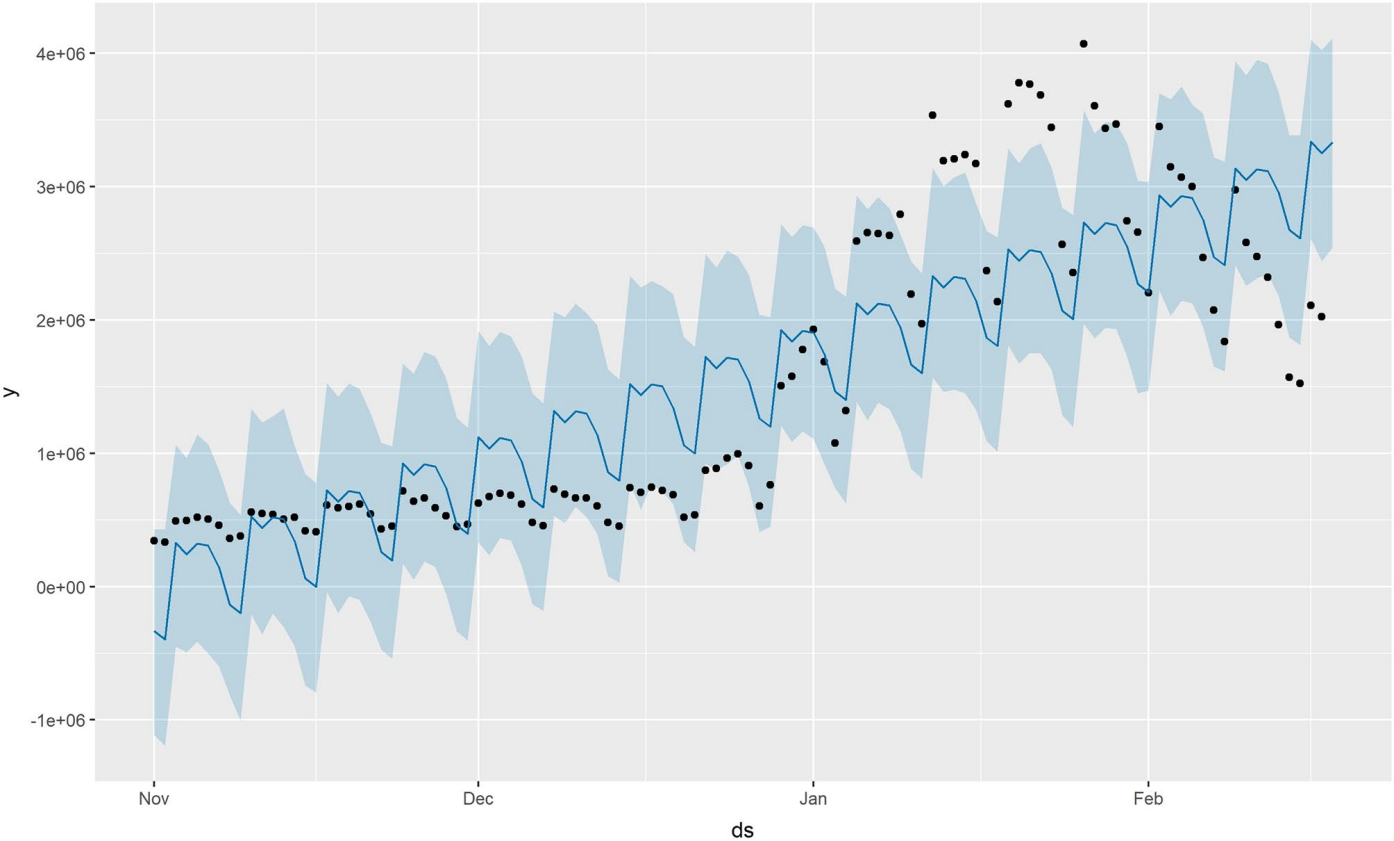
Prediction performance of the Prophet model.

Figure 8 showed the prediction performance of the Prophet model. In Fig. 8, the black dots represent the observed values, the blue line represents the predicted values, and the light-blue areas represent the 95% confidence intervals of the predicted values. As shown in Fig. 8, the predicted values were relatively stable within the 95% confidence interval from November 2021 to January 2022; however, after that, most of the predicted values fell outside the 95% confidence interval. This is due to the fact that the data this time is relatively stable and there are fewer outliers observations.

### Comparison of ARIMA and Prophet models

**Figure.9:**
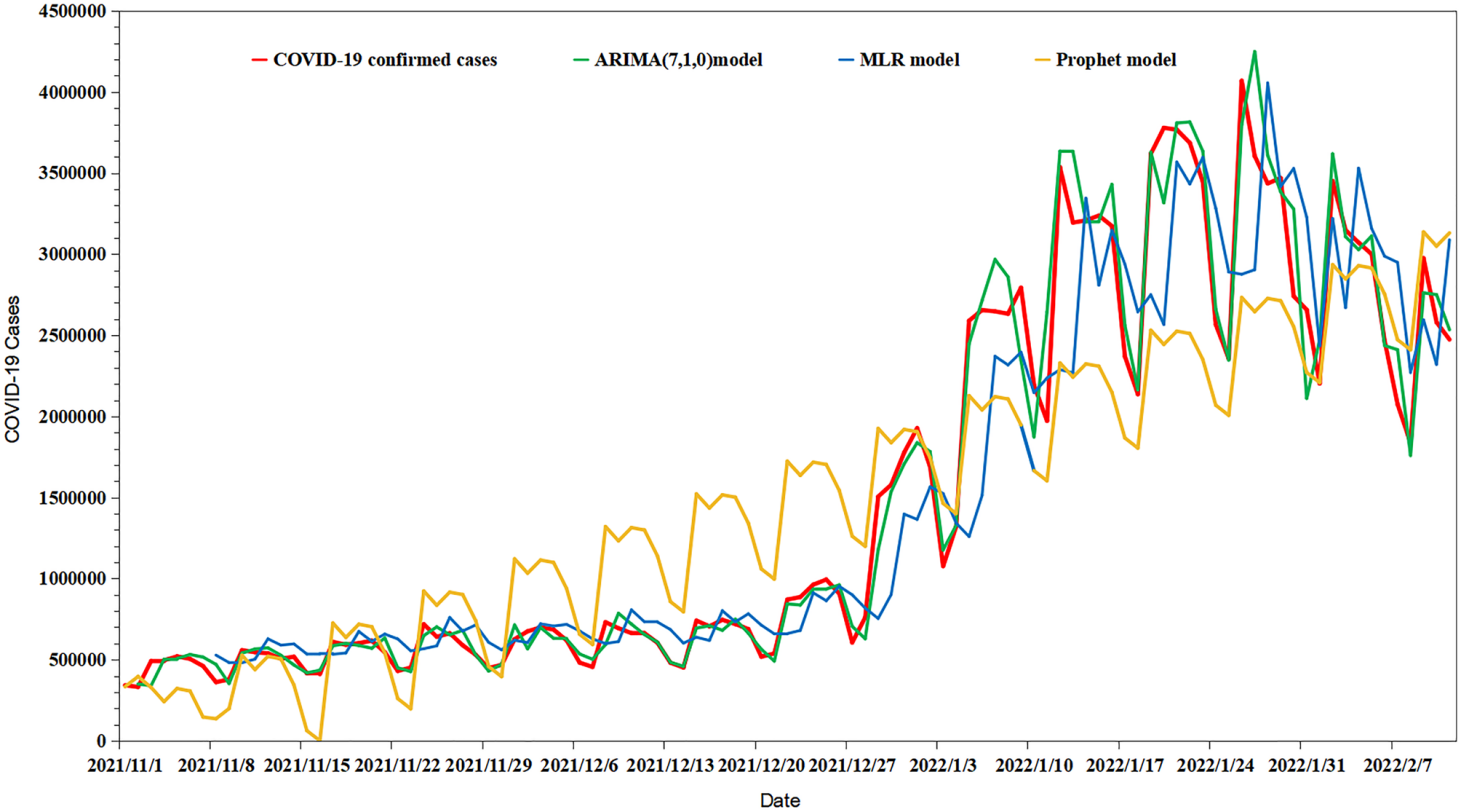
Comparison of prediction performance by ARIMA, MLR, and Prophet models.

As a first-order difference was conducted in the process of constructing the ARIMA (7,1,0) model and the data sliding method was carried out, only 102 observations were available to compare the predicted performances of the ARIMA, MLR, and Prophet models. The predicted and observed values fitted by the three models were used to calculate the MAE, MAPE, and RMSE, which were used to compare the predicted performances in this study. As shown in Table 5, the MAE, MAPE, and RMSE values of the ARIMA(7,1,0) model were lower than those of the MLR and Prophet models both in fitting performance and forecasting performance parts, indicating that the ARIMA(7,1,0) model has superior prediction performance and can be applied for the prediction of daily confirmed COVID-19 cases. The predicted value curve fitted by ARIMA(7,1,0) overlapped with the actual trend of COVID-19 incidence, indicating that ARIMA(7,1,0) was able to simulate the COVID-19 incidence well, and the prediction results were more accurate than those of the MLR and Prophet models (Fig. 9). Therefore, the ARIMA (7,1,0) model was used to perform an extrapolation to predict global daily confirmed COVID-19 cases from February 18 to March 18, 2022 (Table 6).

**Table 5:** The Comparison of MAE, MAPE, and RMSE values of three models.

Evaluating indicator	Fitting performance part			Forecasting performance part		
	ARIMA	MLR	Prophet	ARIMA	MLR	Prophet
MAE	191.45	270,300.04	483,890.1235	183.29	252.72	251.78
MAPE	0.779	0.201	0.452	1.482	2.131	2.01
RMSE	2912.91	2,666,973.70	3,785,828.26	1697.455301	2151.56	2116.99

**Table 6:** Predictions of global daily confirmed COVID-19 cases by ARIMA(7,1,0) model.

Date	Predicted values	95% Lower confidence limit	95% Upper confidence limit
2022/2/18	1,955,794	1,626,346	2,332,598
2022/2/19	1,853,369	1,424,349	2,372,031
2022/2/20	1,603,106	1,158,588	2,163,789
2022/2/21	1,314,136	901,210	1,853,874
2022/2/22	1,286,272	841,850	1,885,660
2022/2/23	1,729,193	1,084,514	2,623,562
2022/2/24	1,673,148	1,008,693	2,619,120
2022/2/25	1,641,240	873,014	2,826,161
2022/2/26	1,581,310	755,366	2,942,926
2022/2/27	1,403,428	608,806	2,791,468
2022/2/28	1,186,792	471,242	2,503,019
2022/3/1	1,177,246	430,439	2,617,044
2022/3/2	1,553,774	525,599	3,623,578
2022/3/3	1,525,385	479,225	3,717,610
2022/3/4	1,524,792	416,056	4,026,490
2022/3/5	1,499,786	358,948	4,249,089
2022/3/6	1,370,077	289,812	4,132,909
2022/3/7	1,198,318	225,406	3,825,370
2022/3/8	1,209,965	203,413	4,066,991
2022/3/9	1,579,760	238,375	5,567,260
2022/3/10	1,580,305	214,813	5,817,731
2022/3/11	1,614,213	186,676	6,333,940
2022/3/12	1,625,062	160,973	6,750,495
2022/3/13	1,530,695	130,617	6,693,222
2022/3/14	1,386,464	102,414	6,350,747
2022/3/15	1,429,094	91,766	6,828,348
2022/3/16	1,856,360	104,007	9,218,235
2022/3/17	1,897,444	93,062	9,760,204
2022/3/18	1,983,675	80,963	10,673,383

## Discussion

Globally, with the rapid spread of the Omicron variant, the number of confirmed COVID-19 cases has continued to increase. Many countries are facing severe epidemic trends for this infectious disease. In this study, the global daily confirmed cases of COVID-19 between November 1, 2021, and February 17, 2022, were obtained from the World Health Organization website. The ARIMA, MLR,and Prophet models were applied to forecast the COVID-19 epidemic trends. Our findings showed that the ARIMA, MLR, and Prophet models could be applied to forecast daily confirmed COVID-19 cases; however, the ARIMA model had a superior prediction performance compared to the MLR and Prophet models.

According to the characteristics of the data, distribution, and sample size^38^, choosing a suitable model for daily confirmed COVID-19 cases is a prerequisite for obtaining more accurate prediction results. There were 109 observations in this study, and the sample sizes and data characteristics met the requirements for constructing the ARIMA, MLR, and Prophet models. Moreover, the ARIMA model is a classical time series prediction approach with several advantages for predicting the incidence of infectious diseases^38^. The major advantage of the ARIMA model is that it addresses linear problems that can reveal the dynamic laws between historical and predicted data^20,39^. The ARIMA model considers the trend, periodicity, and randomness of the time series, which can also quantify the expression by virtue of the model parameters^40^. Multiple linear regression models are widely used to predict the incidence of infectious diseases, and have the advantages of simple and fast modeling^31,32^. Rath et al.^32^ used a multiple linear regression model to forecast new active cases of the COVID-19 pandemic, and the model achieved remarkable accuracy in COVID-19 recognition.

The Prophet model, developed by Facebook in 2017, has been widely used in medicine^41^, environment^35^, and biology^42^ in recent years. Compared with traditional time-series forecasting models, the Prophet model has many advantages, such as its ability to consider trends, periodicity, special events, and outlier factors in the modelling process^35^, and its flexibility and simplicity of construction. In addition, the Prophet model has strong generalization capability and performs better in predicting the incidence of infectious diseases^33^. Xie et al.^33^ used the ARIMA and Prophet models to predict the incidence of HFMD, and the results showed that the prediction performance of the Prophet model was better than that of the ARIMA model. Tulshyan et al.^41^ used the Prophet models to forecast COVID-19 positive cases and fatalities in India over a 30-Day, the study showed that the Prophet model performs better in terms of accuracy with real data.

However, in this study, the MAE, MAPE, and RMSE values of the ARIMA(7,1,0) model were lower than those of the MLR and Prophet models both in fitting performance and forecasting performance parts. Our findings proved that the ARIMA model had superior prediction performance compared to the Prophet model, which was the opposite of their findings^33,41^. There are several possible explanations for this finding. First, the sample size of this study was 109, which met the requirements for modeling the ARIMA, MLR, and Prophet models. However, the prophet model is based on time series decomposition and machine learning fitting, which is more suitable for the long-term prediction of large samples and stabilized data^34,35^. Second, the MLR model has some disadvantages concerning its practical application^43^. For example, it tends to over fit when noisy data are used^43^. When outliers and influential observations are used to build MLR models, the accuracy of their predictions decreases. The prophet method was initially developed to address business-related issues^44^. Third, the time span of the data was from November 1, 2021, to February 17, 2022, which was the period of an outbreak of the Omicron variant. Therefore, the MLR and Prophet models were inferior to the ARIMA model in capturing short-term dramatic changes in the daily confirmed COVID-19 case sequences.

Therefore, we cannot apply predictive techniques blindly to real-world research. In general, data on the incidence of infectious diseases are characterized by linearity, seasonality, periodicity, and randomness^40^. Once the research data were obtained, the characteristics of the data and sample size were observed, and an appropriate predictive model was selected for the prediction. The traditional ARIMA time series forecasting model is well suited, particularly when the information on the research data is insufficient. It can rapidly predict infectious disease epidemics.

Our study has several limitations. First, the ARIMA model specializes in addressing linear problems^12^. However, the nonlinear part of a time series cannot be handled well^27^. Second, there may be the possibility of under-reporting of cases and deaths or delays in notifications, which may also lead to biased results. Third, the predicted values of the global daily confirmed COVID-19 cases from February 18 to March 18, 2022, all fell within the 95% confidence interval, indicating that there were no unexpected outbreaks of the Omicron variant during this period. However, the prevalence of COVID-19 is closely related to meteorological factors^45^, health care factors, and human mobility^35^. Therefore, in future studies, we need to consider the influential factors that affect the occurrence of COVID-19 in the modelling procedures and update the data continuously to obtain more accurate predictions.

## Conclusions

In our study, we collected data on global daily confirmed cases of COVID-19 between November 1, 2021, and February 17, 2022, from the World Health Organization website. ARIMA, MLR, and Prophet models were constructed and compared. The study showed that the ARIMA model had superior prediction performance compared to the MLR and Prophet models. These prediction results can provide reference information for COVID-19 prevention and control worldwide.

## Supplementary Information

Supplementary Information.

## Data Availability

The data used or analyzed during the current study are available from the website of the World Health Organization(https://covid19.who.int/).
